# Do People Dream of Sex with Androids? Neuroscience Behind Cybersex and Technosexual Behaviour

**DOI:** 10.3390/brainsci16101083

**Published:** 2026-10-09

**Authors:** Rafał Gerymski

**Affiliations:** Department of Psychology of Individual Differences, Institute of Psychology, Opole University, 45-040 Opole, Poland; rafal.gerymski@uni.opole.pl

**Keywords:** cybersex, technosexual behaviour, online sexual activity, technology-mediated sexual interactions, digisexuality, sexual interaction in digital contexts

## Abstract

**Highlights:**

**What are the main findings?**

- Cybersex and technosexual behaviour are distinct but related forms of technology-mediated sexual behaviour that should be understood within a biopsychosocial framework.
- These behaviours can be associated with both adaptive sexual expression and problematic use, with psychological factors and reward-related neurobiological mechanisms playing important roles.

**What are the implications of the main findings?**

- Cybersex and technosex should not be inherently pathologised; clinical significance should be determined by distress, impairment, and the context and pattern of use.
- Further research should harmonise definitions and investigate the psychological and neurobiological mechanisms of emerging forms of technology-mediated sexual behaviour, particularly those involving AI, virtual reality, and sex robots.

**Abstract:**

Technology-mediated sexual behaviours, including cybersex and technosex, are becoming increasingly prevalent as digital technologies, virtual reality, artificial intelligence, and interactive devices continue to develop. Although these behaviours may offer numerous benefits, such as facilitating sexual exploration, supporting individuals with disabilities, and enabling long-distance intimacy, they may also contribute to problematic patterns of use in some individuals. This review synthesises current knowledge on the psychological and neurobiological mechanisms underlying cybersex and technosexual behaviour. The article discusses conceptual and definitional issues, epidemiological data, and theoretical frameworks, including the Triple A model and the “quasi-necrophilia” framework, to explain the characteristics and motivations behind technology-mediated sexual activities. Furthermore, it summarises psychological factors associated with both adaptive and maladaptive use, emphasising the role of emotion regulation, personality traits, neurodevelopmental conditions, mental health, and sexual motivations. The review also presents current evidence on the neurobiology of cybersex and technosex, highlighting the involvement of dopaminergic reward pathways and similarities to other reward-related behaviours. Finally, available approaches to the treatment of problematic technology-mediated sexual behaviour are discussed, including pharmacotherapy, as well as psychoeducation and psychotherapy, with particular attention to the importance of addressing underlying psychological conditions. The article argues that cybersex and technosex should not be inherently pathologised—instead, they should be understood within a biopsychosocial framework that distinguishes between healthy sexual expression and clinically significant impairment.

## 1. Introduction

The title of this manuscript is a reference to the Philip K. Dick’s novel titled “Do Androids Dream of Electric Sheep?”, which is the basis for the well-known “Blade Runner” films—a vision of the future that also shows potential changes in human sexuality. People are increasingly using the internet and technology for sexual purposes. With advances in artificial intelligence and technology, these behaviours will become more common. Technology-mediated sexual behaviours such as cybersex and technosex have many advantages and disadvantages—they can be performed anonymously, allow for the fulfilment of sexual desires without the risk of pregnancy, reduce the possibility of contracting sexually transmitted diseases, and allow for the exploration of one’s sexuality without negative consequences. The aforementioned anonymity and the possibility of consequence-free exploration constitute both positive and negative aspects of cybersexuality and technosexuality. Unfortunately, for some individuals, the virtual world can become parallel (or even more important) to the real world. What is more, technology-mediated sexual behaviours can also lead to a reduced sense of responsibility, objectification of a human being, or even raising the threshold of sexual reactivity. Fundamentally, there is nothing wrong with cybersex and technosex, and it is incorrectly pathologised and demonised [1], but in some cases it can lead to negative consequences in one’s life. On the other hand, technology-mediated sexual behaviour might be the only way of fulfilling one’s sexual needs, for example, in individuals coping with various disabilities. Unfortunately, there is a lack of standardisation of the concept of technology-mediated sexual behaviours. What is more, researchers sometimes operate mainly on neurobiological mechanisms and research results, sometimes forgetting about the essence of psychological theories. Therefore, synthesising existing knowledge on the interaction of technology and sexuality is essential for future high-quality research.

## 2. Definitions of Cybersex and Technosex

The definition of technology-mediated sexual behaviours remains unsettled. There are many umbrella terms in the scientific literature that attempt to encompass a range of specific behaviours, from searching for a partner via the Internet to generating pornography using artificial intelligence (AI). McArthur and Twist [2], as well as Desbuleux, Desbuleux and Fuss [3], use the term “digisexuality” to describe a broad range of technologies that mediate sexual experiences, such as sexting, pornography consumption, the use of virtual reality for sexual purposes, or even highly realistic sex dolls or robots [2,3]. This definition seems to combine the use of cyberspace and advanced technology. The most comprehensive classification of various definitions of technology-mediated sexual behaviours and umbrella terms is provided by Döring. She proposes using the terminology “sexual interaction in digital contexts” to describe a wide range of sexual behaviours in cyberspace and with the use of advanced technologies [4,5]. The point of an umbrella term is to group many related phenomena under one single, broad label. This has its advantages and disadvantages. Terms such as “digisexuality” and “sexual interactions in digital contexts” are very inclusive umbrella terms, but the lack of distinction between cybersex and technosex can be problematic. As will be presented in this review, cybersex and technosex differ significantly, as technosexual behaviour can involve a significantly greater number of stimuli, often being a multi-sensory stimulation on haptic, visual and auditory levels [6,7]. This highlights that using umbrella terms in this case may be limiting, and underlines that inclusiveness can lead to the loss of important differences between studied phenomena.

Therefore, what are cybersex and technosex? The terms “cybersex” and “cybersexuality” can encompass many aspects, depending on the definition adopted by cited authors. Griffiths underlines that sexual activities in the online environment encompass a broad range of behaviours, from passive ones (such as watching pornography) to engaging in sexual interactions with other individuals [8]. Cooper’s very broad, universal and now classic definition emphasises that cybersex refers to online behaviours aimed at expressing human sexuality [9,10]. Similarly, Gerymski and colleagues define cybersex as a phenomenon involving the use of cyberspace (e.g., the Internet) to express one’s sexuality and fulfil one’s sexual needs, through behaviours such as searching for a partner via the Internet, sexting (the exchange of sexually explicit messages), pornography consumption, or engaging in cyber sexworking (both as a recipient and provider of such services). Importantly, they underline that cybersex is not the same as technosexual behaviour and that most classic definitions of cybersex do not include phenomena such as the use of AI or sex robots [6,11].

When it comes to the definition of “technosexual behaviour” or “technosex”, Bardzell and Bardzell [12] define them as an interaction between human sexuality and technology. They also underline that it can be defined as an attraction toward technology-mediated activities or just the use of technology for sexual purposes [6,12]. Gerymski emphasises that the terms cybersex and technosex are sometimes used interchangeably, which is incorrect. Technosex can refer to the use of artificial intelligence (e.g., sex chatbots or generative AI), technologically advanced sex toys (also known as “teledildonics”), or even humanoid robots (androids), which are not always used via cyberspace or the Internet [6]. Both cyberspace and advanced technology can be defined from a “digital” perspective, which suggests that the umbrella term of digisexuality is correct. Nevertheless, it is important to understand that cybersex and technosex are distinct phenomena.

## 3. Prevalence of Cybersex and Technosex

The available data indicate that technology-mediated sexual behaviours are relatively common, because data show that the majority of internet users may engage in them [11]. A large sample study (N = 1500) on the German population showed that 68% of its study participants engaged in online sexual activity and 41% of them used sexting. From the studied sample, 55% of the participants engaged in seeking sexuality information online, and 11% were paying for online sexual services [13]. Another large study, again on the German sample (N = 5573 initially, N = 3564 at the final stage), showed that 66% of studied individuals used pornography, around 30% were engaged in sexting, and also around 30% were using dating apps. What is more, 5.3% of the participants admitted to using VR to consume pornography, and 3.9% admitted to using a sex doll or sex robot [3]. Those results underline that technosexual behaviour is not as common as cybersex. Also, meta-analyses on sexting show that it is a fairly common behaviour. Mori and colleagues analysed 50 studies and concluded that around 40–50% of young adults engaged in sexting [14]. Madigan and her team discuss, based on their meta-analysis, that among underage youth, the prevalence is probably much lower—between 15 and 27% [15]. Also, a study on a sample of US citizens (N = 7512) provides interesting information. Results gathered by Gesselman and her team show that around 50% engaged in pornography consumption, 29% admitted to sexting practice, and around 18% declared visiting sex webcam services (which can be considered a form of cyber sexworking consumption) [16].

Unfortunately, data on problematic involvement in cybersex and technosex are limited. A meta-analysis from 2025 on problematic pornography use indicates that this phenomenon affects approximately 13% of individuals [17]. It is important to highlight that the data on non-problematic and problematic engagement in cybersex and technosex are not consistent. Furthermore, there are no epidemiological publications (on sufficiently large and representative samples) on engagement in cyber sexworking, the use of AI chatbots for sexual purposes, or sex robots. What is more, data on problematic technology-mediated sexual behaviour primarily concerns the problematic use of pornography. Nevertheless, the available research shows that non-problematic and problematic cybersex and technosex are more common among men than women, and the percentage of engagement in technology-mediated sexual behaviours varies depending on the study and studied phenomenon [3,13].

## 4. Characteristics of Cybersex and Technosexual Behaviour: The Triple A Model and the “Quasi-Necrophilia” Framework

The Triple A model that explains the engagement in online sexual activity (OSA) was proposed by Cooper [8,18]. It can also be extrapolated to other technology-mediated sexual behaviours [11]. It addresses three characteristics of online behaviour that facilitate cybersex: accessibility, affordability, and anonymity. First, most people use smartphones, which give individuals constant access to the Internet, providing easy access to sexual stimuli. Second, fulfilling sexual needs online is often cost-free or low-cost (both economically and in terms of psychological investment). Third, sexual behaviour via the Internet provides a sense of anonymity (which is actually only apparent, because the user is not anonymous to the Internet service provider). These three characteristics make it easier to overcome barriers in the sexual sphere and can also be a reason to engage in sexual behaviours that people would usually avoid in everyday life—which is both an advantage and a potential disadvantage of sexual behaviours carried out using technology. The Triple A model explains why people may reach for cybersex and technosex, but it also provides an important explanation for potential compulsive behaviours [8,11,18].

To complement the Triple A model, some authors have introduced the theoretical concept of asymmetric control in digital intimacy, controversially termed the “quasi-necrophilia” framework [6,19]. It is vital to clarify that this concept is an unverified theoretical proposal and should not be interpreted as pathologizing digital sexual expression or equating it with paraphilic disorders. Instead, its core theoretical insight concerns the psychological feature of absolute user control and absence of relational risk. This framework shows that individuals might be engaging in certain sexual behaviours (including cybersex or technosex) due to the complete control over the subject of the sexual interaction. This is crucial because in cybersexual and technosexual settings, there are virtually no limits and the chance of causing negative affect in the person involved is very low [6,19]. Technology-mediated sexual behaviour allows for the fulfilment of needs with virtually complete control and minimal consequences. The object of interaction in a cyberspace setting or during the use of technology for sexual purposes (e.g., a sex chatbot or sex robot) will not refuse one’s orders and needs, will not point out one’s negative traits, and will not suffer negative consequences (except, e.g., mechanical damage). On the one hand, not being exposed to rejection can be a good thing—for example, individuals struggling with disabilities will not suffer from rejection or ridicule thanks to engaging in cybersex and technosex. On the other hand, it can lead to avoiding interaction with a real individual or treating them more like a sexual object [6,19].

**It must be explicitly emphasized that the use of term “quasi-necrophilia” can be overly pathologizing, misleading, and improperly implies a link to sexual preference disorders** (**paraphilias**)**.** Therefore, it is worth considering whether researchers might refer to this model using different terminology. Names such as “Asymmetric Control Model” or “Unilateral Intimacy Framework” could prove to be very apt suggestions.

## 5. Psychological Factors and Neurobiological Mechanisms Related to Engagement in the Technology-Mediated Sexual Activities

Multiple factors can lead to non-problematic and problematic use of cybersex and technosexual behaviours. Factors derived from the available scientific literature can be grouped into 3 categories: (1) individual predispositions, (2) mental health and emotion regulation, as well as (3) sexual motivations [11,20,21,22,23]. To enhance the clarity of the narrative, they are presented in Table 1.

The aforementioned factors have deliberately not been presented in terms of positive and negative factors. This is due to two issues. First, these factors may be a source of ethical dilemmas. For example, a person struggling with various disabilities often has no choice but to express their sexuality using the Internet or technology. However, the constant fulfilment of sexual needs in these areas can be consuming, leading to even greater isolation from real life and interpersonal relationships in everyday life [6]. This raises an important ethical issue: should these individuals be supported in expressing their sexuality in this form (as the only potentially available one), despite the potential negative consequences? Second, even potentially negative factors do not necessarily lead to problematic use of cybersex and technosexuality. Also, positive motivations can lead to problematic use. Cybersex and technosexual behaviour, especially while presenting exaggerated and unrealistic stimuli, can be the complete opposite of a face-to-face interaction with a real individual, during which the boundaries and needs of the other person must be respected. Control over the object and the ability to freely create the fulfilment of sexual needs through cybersex and technosex may be what encourages some of their users to engage more in these behaviours. Importantly, the more cybersex and technosexuality deviate from everyday life and realistic patterns, the more problematic it can be. On the other hand, cybersexual behaviours resembling real life can be a healthy addition to our sex lives and do not necessarily pose a health risk. For completeness, four examples of technology-mediated sexual behaviors can be discussed: (1) consensual sexting with a partner, (2) occasional use of pornography that shows people and behaviors similar to ourselves, which are highly realistic and are based on respecting the boundaries and needs of all people involved, (3) use of pornography that depicts exaggerated, unrealistic stimuli, and (4) use of child-like sex robots, with which the user can do whatever one likes, without respecting boundaries or even the law. This emphasises that the quality and type of cybersexual and technosexual behaviours we engage in are important, and that cybersex and technosexuality themselves should not be demonised, as the problem may lie in the purposes for which, and the ways in which, we engage in them.

It is extremely interesting in the case of sex robots usage. Unfortunately, the state of research using sex robots is worrying, as there is a lack of good-quality data, and the vast majority of available publications are theoretical articles [24]. However, studies on sex doll users can provide some guidance on the possible consequences of the use of androids for sexual purposes. A study on doll owners (N = 217) analysed two sub-groups of individuals: (1) who treated the doll as a sex toy, and (2) who treated the doll as a partner. The results suggest that the tendency to anthropomorphise dolls can be associated with negative attitudes toward women [25]. A different study (N = 214) on doll users concludes that sex dolls may be used as an outlet for illegal sexual preferences, such as pedophilia [26]. These studies emphasise that android sex robots should be used with caution. However, it is impossible to indicate the direction of this relationship—whether the use of dolls leads to socially undesirable behaviours, or whether some individuals use dolls as a way to fulfil their illegal needs. This requires further research. The use of sex robots can have positive effects, for example, when working with the elderly or those struggling with disabilities [6,27]. A modest study on physicians and therapists (N = 72) shows that around half of the professionals believe that sex robots can be used in sexual therapy [28]. Therefore, due to potential negative consequences, the implementation of sex robots in sexual rehabilitation must be done with great care.

Preliminary evidence suggests that the neurobiological mechanisms underlying technology-mediated sexual behaviors can involve neural circuits similar to those engaged during offline sexual activity and other reward-driven behaviors. However, it is important to emphasize that direct neuroimaging evidence is currently restricted almost exclusively to problematic pornography use, and cannot be directly extrapolated as proven fact to technosexual behaviors (such as interactions with AI, virtual reality, or sex robots). It is possible that rather than operating through distinct mechanisms, technology-mediated sexual behaviors may differ in the degree and pattern of dopaminergic engagement due to multisensory and immersive stimuli, though this remains an empirical hypothesis requiring direct neuroscientific investigation. In essence, the difference between everyday sexual behaviour and technology-mediated sexual behaviour is that cybersex and technosex often provide instant gratification without the need for investment of resources. It is important because intensive stimulation of the reward system can lead to changes in neural plasticity, affecting one’s cognitive processes, emotion regulation and reward processing [23].

Neuroscience research has provided important insights into the neural mechanisms potentially underlying technology-mediated sexual behaviours, although the available evidence is currently limited and derives predominantly from studies of problematic pornography use [29,30,31]. Thus, the involvement of neural systems implicated in reward and sexual behaviour in cybersex and technosex remains a plausible hypothesis rather than an established finding. The mesolimbic pathway consists primarily of dopaminergic neurons originating in the ventral tegmental area (VTA) that project to the ventral striatum, a subcortical brain structure involved in reward and motivation processing, particularly the nucleus accumbens (NAc), its major target region. Research on sexual and erotic stimuli suggests that this circuitry is involved in reward-related responses to such stimuli [32,33]. However, whether and to what extent these mechanisms are similarly engaged during technology-mediated sexual behaviours remains to be established through direct investigation.

Important evidence regarding the neurobiological mechanisms potentially relevant to cybersex comes from studies of individuals who problematically use pornography. In an fMRI study comparing responses to erotic stimuli between a control group (N = 24) and individuals with problematic pornography use (PPU; N = 28), Gola and colleagues observed increased ventral striatal activation in the PPU group [30]. In that study, significant group differences were observed in responses to cues predicting erotic stimuli, but not in responses to the erotic stimuli themselves, suggesting a cue-reactivity pattern similar to that observed in some addictive behaviours.

On the other hand, Kühn and Gallinat studied a small sample of pornography users (N = 64). They found a negative relationship between pornography consumption frequency and the volume of the grey matter in the right caudate, as well as left striatum activation during sexual cue–reactivity, which play a significant role in emotion regulation and reward processing [34]. It is important to note that, because this finding relies on a cross-sectional design, no causal inferences can be drawn, as the observed changes may represent either a pre-existing neurobiological vulnerability predisposing individuals to frequent use or, conversely, a neural adaptation resulting from extensive consumption.

Moreover, available data show that oxytocin and vasopressin can also play a role in cybersexual behaviour. A neuroscientific study that included a control group (N = 53) and individuals using pornography problematically (N = 69) concludes that the aforementioned hormones responsible for creating and maintaining bonds between people may be linked to problematic pornography use [35]. They also suggest that oxytocin, vasopressin, and symptoms of mental disorders may contribute to reduced empathy, which in turn may contribute to the development of problematic pornography consumption and compulsive sexual behaviours [35]. However, the described cross-sectional case–control study reflects observed associations rather than a proven longitudinal developmental pathway. Another fMRI study was performed on a group of young men (N = 55) with the use of oxytocin administration. During the fMRI, participants were shown two types of scenes: erotic situations and social situations designed to evoke fear. The results showed that when participants viewed erotic images, oxytocin increased activity in brain areas involved in the reward and dopamine systems [36].

Unfortunately, most neuroscientific data concern pornography use, and the number of publications is limited. There is a lack of data on technosexuality, i.e., the use of robots, virtual reality, or artificial intelligence for sexual purposes. It is possible that technosex can be more dopaminergic than cybersex. Sex robots or the use of virtual reality provide multi-sensory stimulation on haptic, visual and auditory levels [6,7]. It makes the VR-related and robot-related sexual experiences very immersive, which can potentially engage one’s brain more extensively than cybersex activities. Sex robots, while still not very common, also pose a potential threat. They can provide a substitute for human contact, thus fulfilling a range of sexual and non-sexual needs of their users [26]. Based on the Media Equation Theory [37], it can be concluded that people have a tendency to attribute human characteristics to computers and robots. On the other hand, according to the Uncanny Valley Hypothesis, it should be recognised that too much similarity between AI or a robot and a human can have the opposite effect [38]. This makes some examples of technosex more similar to real-life sex in comparison to different forms of technology-mediated sexual activities. It poses a significant risk, as taking into account the many advantages of technosex (see the Triple A model and the “quasi-necrophilia” framework presented earlier), it may cause some individuals to prefer contact with a sex robot over a real person. What is more, cybersex and technosexual behaviour sometimes involves exaggerated, intense stimuli. It is hypothesized that they can stimulate our reward system more strongly than offline sexual behaviour and those without the use of technology [39]. Therefore, studies on technosexual behaviour and its implementation in sexual rehabilitation need to take into account these potential threats.

## 6. Pharmacotherapy and Psychological Help in Problematic Use of Cybersex and Technosex

As indicated earlier, individuals may express their sexuality through technology or cyberspace for many positive reasons, e.g., to diversify their sex lives or to combat their own limitations resulting from disability. Unfortunately, cybersex and technosex can sometimes become overwhelming, causing significant suffering for the individual or their surroundings. Therefore, it is necessary to outline the potential forms of help for individuals who use these behaviours in a problematic way. Treatment of problematic use of cybersex and technosex can be categorised into two categories: (1) causal treatment or (2) symptomatic treatment. These categories can also be divided into medical interventions and psychological help or psychotherapy. It is impossible to say that one type of treatment is better than another—it all depends on the individual case. These methods often complement each other and can be used in parallel—the same as with other mental health problems, such as depression.

In instances where engagement in cybersex or technosexual behavior becomes problematic or compulsive, pharmacotherapy is not directed at eliminating those behaviors itself, but rather at managing diagnosed comorbid conditions (e.g., major depressive disorder, ADHD) or addressing symptoms of underlying Compulsive Sexual Behavior Disorder (CSBD). For example, patients using cybersex problematically due to depression can undergo pharmacotherapy based on SSRIs (selective serotonin reuptake inhibitors), SNRIs (serotonin–norepinephrine reuptake inhibitors), TCAs (Tricyclic Antidepressants) or MAOIs (Monoamine Oxidase Inhibitors). On the other hand, individuals overusing cybersex and technosex due to failure to cope with one’s neurodevelopmental conditions (e.g., ADHD) may reach for medications based on MPHs (methylphenidate), ATXs (atomoxetine), or LDXs (lisdexamfetamine) [40]. Problematic cybersex and technosex can also be a manifestation of compulsive sexual behaviour disorder (CSBD). Pharmacotherapy is mostly used only in moderate and severe cases of CSBD, and it includes SSRIs and ORAs (opioid receptor antagonists) [29]. Additionally, emerging literature discusses the theoretical potential of GLP-1 receptor agonists (semaglutide) in modulating reward processing and behavioral compulsions [41,42,43]. However, this remains an unverified, preliminary hypothesis based on early observational and social media data, and must strictly not be interpreted as a clinical treatment recommendation.

The use of pharmacotherapy alone may sometimes be insufficient. It is recommended that it be supplemented with appropriate psychoeducation, psychological support, or psychotherapy. As mentioned earlier, problematic use of cyberspace and technology for sexual purposes may be a symptom of CSBD. If the problematic cybersex and technosex engagement is a result of a compulsive sexual behaviour disorder, individual and group therapies are often recommended [29,44]. Hungarian 3-year longitudinal study on a large sample of young adults (N = 782) shows that compulsive sexual behaviour is probably more state-like than trait-like [45]. In other words, it might not be a stable feature, but a phenomenon that appears under specific conditions. This means that the overall well-being and mental health of individuals who use the internet and technology for sexual purposes in impulsive and problematic ways should be addressed first. This is because the aforementioned problematic use may manifest itself in moments of loneliness, boredom, anxiety, stress or depressive states. In such cases, symptomatic treatment may not be effective and potential risk factors should be addressed first, indicating the importance of causal treatment. The meta-analysis on psychotherapy for problematic pornography use shows that cognitive behavioural therapy (CBT) and acceptance and commitment therapy are effective forms of treating problematic cybersex and related problems [46].

## 7. Conclusions and Limitations

The presented review provides important conclusions. First, definitions of technology-mediated sexual behaviours remain unsystematised and require further harmonisation. Second, one should not analyse cybersexuality and technosexuality without considering both its components: the psychological and neurobiological sphere. Thirdly, technology-mediated sexual behaviour should be analysed from both the recreational and positive, as well as the problematic and negative points of view—relying on one of them may lead to the desensitisation of the essence of the phenomenon or its excessive pathologisation. Finally, conclusions regarding the neurobiology and psychological impact of technosex must remain tentative, given that the available literature relies heavily on observational data, cross-sectional designs, and indirect evidence derived from pornography studies.

The presented review manuscript can be valuable for future psychological and neuroscientific research on cybersexuality, but it is not free from the limitations of unsystematised review articles. The most significant limitations of this narrative review include the risk of publication selection, because the selection of studies can be dependent on the author’s subjective assessment and may favour the inclusion of results consistent with the adopted narrative. Another limitation is the lack of a systematic and comprehensive literature search, which increases the risk of missing important studies. Furthermore, a narrative review is characterised by limited transparency and reproducibility, as the method of literature search, selection, and interpretation may not be sufficiently detailed, and different researchers may obtain a different set of publications and, consequently, different conclusions. The last limitation is the current state of research—problematic technology-mediated sexual behaviour focuses mainly on problematic use of pornography, and there is a lack of research on other aspects of it.

## 8. Future Directions

The definitions and theoretical framework presented in this review provide a basis for further systematic investigation of the subject of cybersexuality and technosexuality. A systematic review could help to structure the available evidence more comprehensively and to verify, refine, or challenge the conclusions presented in this manuscript. However, conducting a meta-analysis may prove challenging because many aspects of technology-mediated sexual behaviours are assessed using diverse and non-standardised measures, with problematic pornography use being a notable exception. Furthermore, given the rapid pace of technological development, the evidence base is likely to evolve considerably in the near future. Increasing access to digital technologies, their ongoing advancement, and the growing body of related research highlight the need for periodic updates of the current review to ensure that the conclusions remain relevant and reflect the most recent evidence.

## Figures and Tables

**Table 1:** Examples of factors related to engagement in the technology-mediated sexual activities.

**1.** **Individual predispositions, such as personality traits, temperament and individual differences**	**Suggested read**
Neurodevelopmental conditions (particularly, individual differences in the functioning of dopaminergic systems)	[20]
High openness to experience and novelty-seeking	[11,20,22]
High neuroticism (susceptibility to negative emotions)	[20,22]
High impulsivity and low self-control	[20,22,23]
**2.** **Mental health and emotion regulation**	
Using technology (e.g., VR or avatars) to build self-confidence in interactions, which later translates into better functioning in real life	[20,22]
Negative self-assessment: low self-esteem and body image perception problems	[20,22]
Sexual behaviour as an **occasionally used form of coping** for relieving tension, negative emotions or reducing stress	[20,21,22]
Sexual behaviour as a **long-term and most frequently used (maladaptive)** form of coping for relieving tension, negative emotions or reducing stress	[20,22,23]
Ill-being—high levels of perceived stress, anxiety symptoms and depressive symptoms	[20,23]
Treating cybersex and technosex as planned and conscious forms of pleasure	[11,21]
Maintaining a long-distance relationship	[11,21]
Coping with relationship conflicts	[11,22]
Personality disorders (e.g., Borderline Personality Disorder)	[20]
Substance use disorders, behavioural addictions and impulse control disorders (such as Compulsive Sexual Behaviour Disorder)	[20,23]
Maladaptive reaction to lack of stimulation, such as perceived boredom	[20,22,23]
Perceived loneliness	[11,20,22]
**3.** **Sexual motivations**	
Fulfilling one’s sexual needs (related to one’s sex drive)	[11,21]
Diversification of sexual life (in the life of an individual and a couple)	[11,21]
Sexual exploration	[11,21]
Education and self-awareness	[11,21]
Sexual enhancement	[11,21]
Occurrence of sexual dysfunctions	[20,22]
Fulfilling sexuality using technology and cyberspace due to limitations (e.g., disability or sexual dysfunctions)	[11]
Sexual preference disorders (e.g., paraphilic disorders, the fulfilment of which in everyday life is difficult, impossible or illegal)	[20]

## Data Availability

No new data were created or analyzed in this study. Data sharing is not applicable to this article.
